# Characterization of an obese population: a retrospective longitudinal study from real-world data in northern Portugal

**DOI:** 10.1186/s12875-023-02023-7

**Published:** 2023-04-15

**Authors:** Rosália Páscoa, Andreia Teixeira, Teresa S. Henriques, Hugo Monteiro, Rosário Monteiro, Carlos Martins

**Affiliations:** 1 Faculty of Medicine, Department of Community Medicine, Information and Health Decision Sciences (MEDCIDS), University of Porto, Al. Prof. Hernâni Monteiro, 4200 - 319 Porto, Portugal; 2 University of Porto, Centre for Health Technology and Services Research (CINTESIS), Porto, Portugal; 3 Administração Regional de Saúde do Norte IP, Health Centre Grouping Porto Ocidental, Family Health Unit Homem do Leme, Porto, Portugal; 4 Instituto Politécnico de Viana do Castelo (IPVC), ADiT-LAB, Viana do Castelo, Portugal; 5 Studies and Planning Department, Administração Regional de Saúde do Norte IP, Porto, Portugal; 6 #H4A Primary Healthcare Research Network, Porto, Portugal

**Keywords:** Primary care, Obesity, Preventive medicine, Real-world data

## Abstract

**Background:**

Obesity is a serious and largely preventable global health problem. Obesity-related electronic health records can be a useful resource to identify and address obesity. The analysis of real-world data from T82-coded (International Classification of Primary Care coding, for obesity) primary care individuals can be an excellent national source of data on obesity’s prevalence, characteristics, and impact on the National Health Service.

**Methods:**

Retrospective longitudinal study, based on a database of electronic medical records, from the Regional Health Administration of northern Portugal. The study objectives were to determine the prevalence of obesity and to characterize an adult obese population in northern Portugal from a bio-demographic point of view along with profiles of comorbidities and the use of health resources. This study used a database of 266,872 patients in December 2019 and screened for diagnostic code T82 from the International Classification of Primary Care.

**Results:**

The prevalence of obesity was 10.2% and the highest prevalence of obesity was in the 65–74 age group (16.1%). The most prevalent morbidities in patients with obesity as coded through ICPC-2 were K86 (uncomplicated hypertension), T90 (non-insulin-dependent diabetes), and K87 (complicated hypertension). Descriptive information showed that T82 subjects used more consultations, medications, and diagnostic tests than non-T82 subjects.

**Conclusions:**

Routine recording of weight and height deserves special attention to allow obesity recognition at an early stage and move on to the appropriate intervention. Future work is necessary to automate the codification of obesity for subjects under 18 years of age, to raise awareness and anticipate the prevention of problems associated with obesity.

Practical strategies need to be implemented, such as the creation of a specific program consultation with truly targeted approaches to obesity.

**Supplementary Information:**

The online version contains supplementary material available at 10.1186/s12875-023-02023-7.

## Introduction

Obesity is a serious and largely preventable global health problem. The worldwide prevalence of obesity nearly tripled between 1975 and 2016 with 13% of the world's adult population (11% of men and 15% of women) obese in 2016 [1].

This problem is increasingly impactful: Scientific evidence shows that the risk for non-communicable diseases increases with an increase in body mass index (BMI). Thus, obesity is a major risk factor for non-communicable diseases including cardiovascular diseases (mainly heart disease and stroke); diabetes; musculoskeletal disorders (especially osteoarthritis); and some cancers (including endometrial, breast, ovarian, prostate, liver, gallbladder, kidney, and colon cancers) [1–3].

Obesity is defined as a BMI greater than or equal to 30 kg/m^2^ [4]. The BMI provides the most useful population-level measure of obesity [1] A 2015 Portuguese survey of adults aged 25–74 years described a national prevalence of obesity of 28.7% (95%CI: 26.8–30.6) and 28.2% (95%CI: 26.4–30.5) in northern Portugal [5].

Globally, there appear to be two main causes for obesity: an increased intake of energy-dense foods that are high in fat and sugars and an increase in physical inactivity due to the increasingly sedentary nature of many work forms, changing modes of transportation, and increasing urbanization [1]. Effective interventions, including those addressing lifestyle in the domains of diet and physical activity that successfully reduce the population's BMI by 1% or 5%, have a significantly positive impact on decreasing obesity-associated comorbidities such as cardiovascular disease, type 2 diabetes, and some types of cancers [6].

Obesity-related electronic health records (EHRs) can be a useful resource to identify and address obesity [3, 7–10]. In Portugal, after the primary health care reform carried out from 2005 with the implementation of the pay-for-performance system, EHRs—including the codification through the International Classification of Primary Care (ICPC-2)—gradually began to be used in a generalized manner [11–13].

The ICPC-2 is a coding system proposed by the World Organization of Family Doctors classification group and includes clinical practices and family physicians [14] Classification with one-letter and two-digit alphanumeric codes is globally organized into chapters based on anatomical systems over etiology [15] According to ICPC-2, code T82 is an obesity diagnosis [13, 14]. The Portuguese EHRs replaced almost all paper data records and simultaneously began monitoring multiple indicators (*e.g.* performance, quality of care, healthcare expenses) through Microsoft power BI® reports [16]. In this context, the analysis of real-world data from T82-coded primary care individuals can be an excellent national source of data on obesity’s prevalence, characteristics, and impact on the National Health Service (NHS).

This study of adults in northern Portugal was based on real-data EHRs and aimed: i) to determine the prevalence of obesity in 2019; and ii) to characterize the obese population from the bio-demographic point of view including a profile of comorbidities and the use of health resources.

## Methods

### Study design and setting

This was a retrospective longitudinal study conducted using real-world data from the northern Portugal regional health administration database in 2019. The Portuguese National Health Service offers universal coverage and is administratively divided into five regions: North, Centre, Lisbon and Vale do Tejo, Alentejo, and Algarve. The entire population of mainland Portugal is registered in one of these administrative health regions according to the geographical area of ​residence. The system organizes and provides all health care (primary and secondary) to that population.

### Data collection

In this study, the Studies and Planning Department of the regional health administration of northern Portugal (Ministry of Health, Portugal) built a database containing the last record available for the following variables for each individual with ICPC code T82: gender, age, weight, height, abdominal perimeter, alcohol intake, smoking status, cholesterol (total, and high-density lipoprotein cholesterol (HDL-C), triglycerides, and systolic and diastolic blood pressure. For each individual, a list of comorbidities was also collected, with the coding date of each of the health problems.

Data regarding the total number of patients with T82 on the list of health problems as well as the population registered in the regional health administration of northern Portugal were also obtained from the regional health administration of northern Portugal. We note the immediate and autonomous coding of the T82 by the Portuguese electronic information system (SClínico®). This continues as long as the individual has a consultation in which he/she is evaluated in terms of weight and height. This approach was established with version 2.7 of SClínico® in March 2019.

Data, referred to until this point, were obtained without aggregation at the individual level for general characterization analysis.

Concerning the data collection related to the use of health resources, the Department of Studies and Planning of the regional health administration of northern Portugal integrated a grouped data section into the database. These data, grouped and organized by age groups through Microsoft Power BI® reports, refer to medical consultations, medications, diagnostic tests, and sick days with absence from work. All individuals aged 18 years or over were considered, taking into account the following age groups: [18–30[, [30–40[, [40–50[, [50–60[, [60–70[, and [80 > [ years old.

Data processing was done through the Department of Studies and Planning of the regional health administration of northern Portugal. The data were extracted from the server through an anonymized data processing and editing platform and delivered securely to the principal investigator following legal regulations and appropriate approval. The database was exported to Microsoft Excel 2016®, and the statistical analysis was performed using SPSS Statistics 25.0® and R software.

### Data cleaning

Initially, data from 421 126 individuals were presented corresponding to all subjects with the ICPC-2 code T82 in 2019 from northern Portugal. The most robust methodological strategy to clean and improve the quality of this real-world database was to define the maximum and minimum values for the weight and height of participants. Thus, it was essential to have representative data of the Portuguese population such as those obtained through the first Portuguese Health Examination Survey (INSEF) [5]. The INSEF was a cross-sectional population-based study representative at a regional and national level. Individuals between 25 and 74 years old and residing in Portugal were selected from the national health users' registry via multi-stage stratified probabilistic sampling. European health examination survey (HES) procedures were followed [17].

Thus, all those outside the 25–74 age group were excluded. Then, all individuals who presented height values outside the minimum of 116.2 cm and the maximum of 194 cm were excluded. And, subsequently, those with weight values ​​outside the minimum of 37.9 kg and a maximum of 160 kg were also excluded [5, 17].

Both previous and new obesity diagnoses were included, and the database included both incident and prevalent individuals. Through the data cleaning performed, individuals who still had the T-82 coding (probably performed at an earlier stage in time and therefore still manually), but who no longer met the criteria for obesity (according to their most up-to-date anthropometric data in the clinical file) were also excluded.

**Fig. 1:**
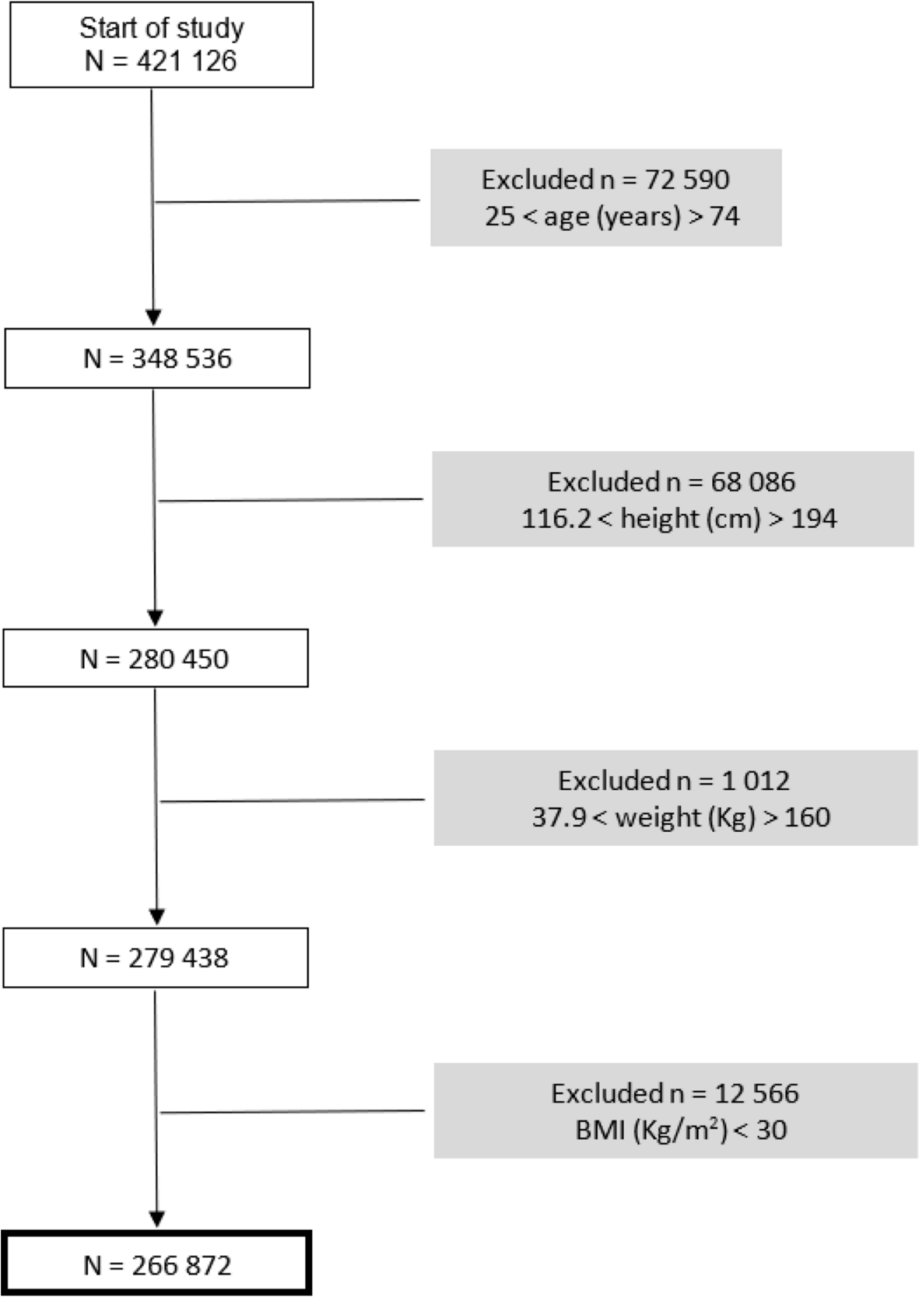
Results from the data cleaning process. BMI: Body Mass Index

After data cleaning process (Fig. 1.), the database allowed the analysis: 1) at the individual level (bio-demographic characterization, with a profile of comorbidities), including the individuals between 25 and 74 years old; 2) at the group level (use of health resources characterization), including the individuals from 18 years old onward.

### Statistical analysis

For data analysis and interpretation purposes, patients with obesity were divided into three categories: class I (BMI 30 to 34.9 kg/m^2^), class II (BMI 35 to 39.9 kg/m^2^), and class III (BMI greater than or equal to 40 kg/m^2^) [4]. The prevalence of obesity was calculated for the adult population (25–74 Years) registered in the regional health administration of northern Portugal in 2019.

The subjects were characterized in terms of health resource utilization via data analysis in Microsoft power BI® reports. These were available by age group and compared individuals with and without T82 coding. Differences concerning the number of patients with consultations (usage rate of medical consultation), the number of patients with medication prescriptions, and the number of patients with diagnostic tests (any medical test carried out to complement diagnosis or to diagnose a condition) were evaluated. Data regarding the usage intensity quotients of medical consultation [number of consultations/number of patients], usage intensity quotients of medication [number of packages/number of patients], intensity quotients of the amount spent on medication (euro) [amount spent in euro on medication/number of patients], diagnostic tests intensity quotients [number of diagnostic tests/number of patients], quotient of medically justified absences from work [number of absences/number of patients], and intensity quotient of sick days [number of sick days/number of patients] were also analyzed. No statistical testing was used to compare subjects with or without T82 coding, and descriptive information only is reported.

The categorical variables were described using absolute and relative frequencies, n (%). The normally distributed continuous variables were described by the mean and respective standard deviation, mean ± SD, and by the minimum (min) and maximum (max) values. In the case of continuous variables not normally distributed, the data were presented by the median and respective interquartile interval (Med (Q_1_; Q_3_)) where Q_1_ is the first quartile and Q_3_ is the third quartile. The normality was verified by observing the histograms.

### Ethics approval

The study protocol was approved by the Health Ethics Committee of the Regional Health Administration of northern Portugal [36/2019 | DEP/2020/81].

## Results

Initially, data from 421 126 individuals were presented. Subsequently, all those outside the 25–74 age group were excluded leaving the database with 348 536 individuals. Next, all individuals who presented height values outside the minimum of 116.2 cm and the maximum of 194 cm were excluded leaving 280 450 individuals. Those with weight values ​​outside the minimum of 37.9 kg and a maximum of 160 kg were then excluded for 279 438 individuals remaining. Finally, BMI values were calculated, and 12 566 with a BMI value < 30 kg/m^2^ were excluded (Fig. 1.). Thus, data from 266 872 individuals were analyzed.

### Prevalence of obesity

**Fig. 2:**
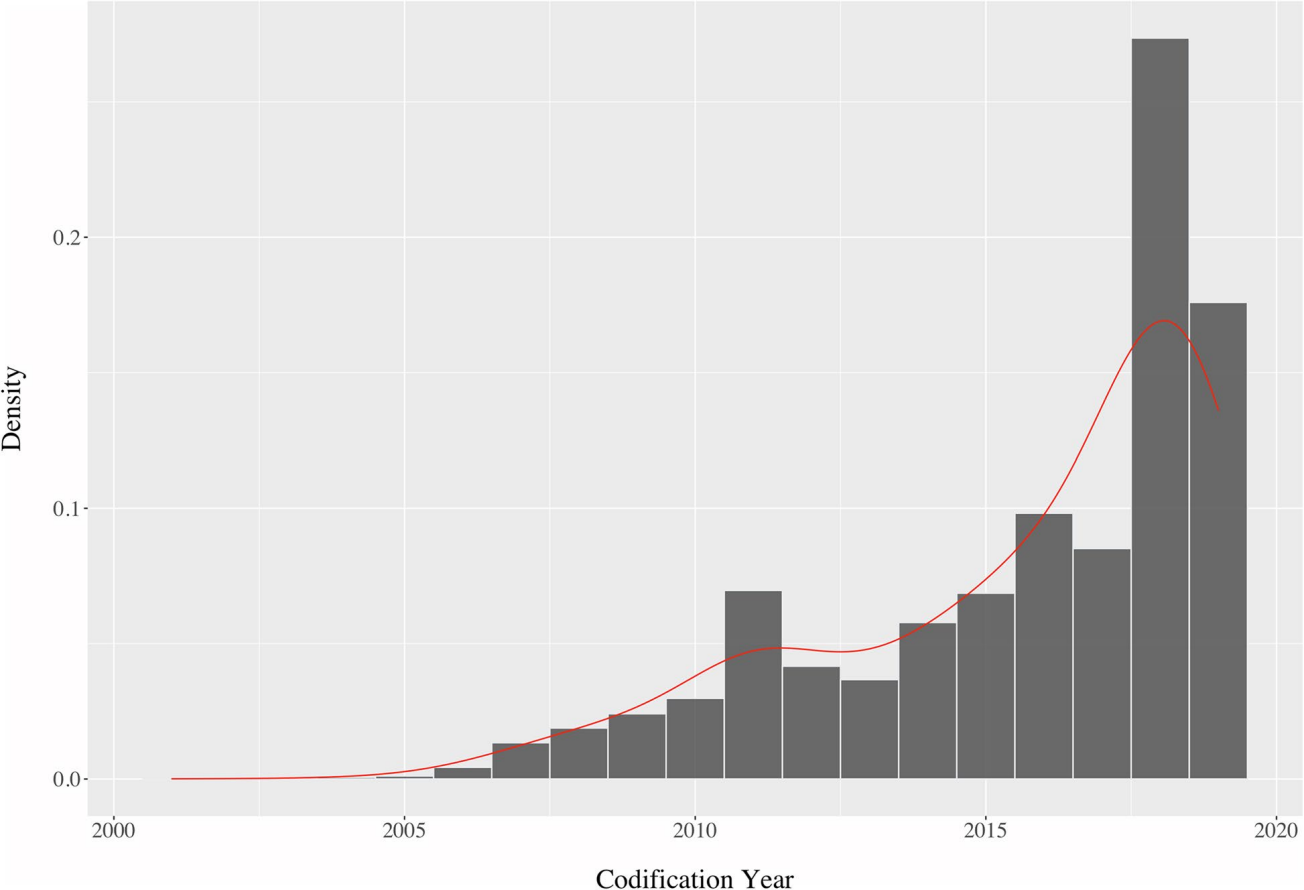
The density of obesity codification over time

A total of 266 872 individuals were identified with a diagnosis of obesity (ICPC-2 code T82) in northern Portugal. The prevalence of obesity obtained in this study was 10.2% considering the 2 619 161 adults aged 25–74 years registered in 2019 in the regional health administration of northern Portugal. The prevalence by age group was higher in groups 65–74 (16.1%) and 55–64 (13.2%) (Table 1.). The T82 ICPC-2 coding of obesity has increased in recent years especially in 2018 (Fig. 2).

**Table 1:** General characterization of the individuals with an obesity diagnosis in northern Portugal identified by ICPC-2 code T82—Obesity. *N* = 266 872

		**Missing**	**Out-of-range values**
**Age** (years), M ± SD, min–max	54.4 ± 12.5, 25–74	0	0
**Age group**, n (%)			
*25–34*	21 442 (8.0)	0	0
*35–44*	40 528 (15.2)	0	0
*45–54*	63 427 (23.8)	0	0
*55–64*	72 698 (27.2)	0	0
*65–74*	68 777 (25.8)	0	0
**Age group prevalence, %**			
*25–34 (n* = *459 497)*	4.7	0	0
*35–44 (n* = *576 009)*	7.0	0	0
*45–54 (n* = *607 030)*	10.4	0	0
*55–64 (n* = *550 671)*	13.2	0	0
*65–74 (n* = *425 954)*	16.1	0	0
**Gender**, n (%)			
*Females*	172 834 (64.8)	0	0
*Males*	94 038 (35.2)	0	0
**Weight** (*kg*), M ± SD, min–max	89.97 ± 13.16, 46–160	0	0
**Height** (*m*), M ± SD, min–max	1.62 ± 0.09, 1.17–1.94	0	0
**Abdominal circumference** (c*m*), M ± SD, min–max	108.2 ± 10.4, 59–195	91 942 (34.5)	[57.5; 197] 344
**Alcohol intake**^a^ (g/week), M ± SD, min–max	61.3 ± 119.3, 0–6020	7 160 (2.7)	__
**Smoking status**^a^ (number of cigarettes/day), M ± SD, min–max	1.4 ± 39.7, 0–20,103	6 148 (2.3)	__
**Total cholesterol** (mg/dl), M ± SD, min–max	187.9 ± 36.9, 28–414	26 224 (10.9)	__
**HDL-C** (mg/dl), M ± SD, min–max	50.8 ± 13.1, 17–168	29 084 (12.2)	__
**Triglycerides** (mg/dl), M ± SD, min–max	136.1 ± 78.8, 17–1673	27 214 (10.2) (2630 > 400 mg/dl)	__
**LDL-C** (mg/dl), M ± SD, min–max	110.1 ± 32.2, -38–330.2	33 302 (14.2)	__
**Systolic blood pressure** (mmHg), M ± SD, min–max	132.1 ± 14.4, 88–224	892 (0.3)	[87.5; 224] 264
**Diastolic blood pressure** (mmHg), M ± SD, min–max	79.9 ± 9.3, 44–128	885 (0.3)	[43.5; 128] 227
**BMI**, n (%)			
*Obesity class I*	186 742 (70.0)	0	0
*Obesity class II*	59 510 (22.3)	0	0
*Obesity class III*	20 620 (7.7)	0	0
**Comorbidities** (in addition to obesity), n (%)			
*0*	104 484 (39.1)	0	0
*1*	101 485 (38.0)	0	0
*2*	50 175 (18.8)	0	0
*3*	8 602 (3.2)	0	0
*4*	1 787 (0.7)	0	0
*5*	287 (0.1)	0	0
*6*	47 (0.0)	0	0
*7*	5 (0.0)	0	0

M ± SD: mean and respective standard deviation; min: minimum; max: maximum

*HDL-C* high-density lipoprotein cholesterol, *LDL-C* low-density lipoprotein cholesterol, *BMI* body mass index

^a^self-reported data (rest are measured data)

### Bio-demographic characterization:

The average age was 54.4 years (range from 25 to 74 years) and 172 834 (64.8%) were women. The largest number of individuals, 72 698 (27.2%), were 55–64 years. Among patients with obesity, 186 742 (70.0%) had class I obesity.

Of the 266 872 individuals, 162 388 (60.9%) had at least one morbidity in addition to obesity. Table 1 provides a more detailed characterization of these individuals.

Those in the 60–70 and 70–80 age groups were most likely to have obesity —18.9% (supplemental material 1).

### Profile of comorbidities

**Fig. 3:**
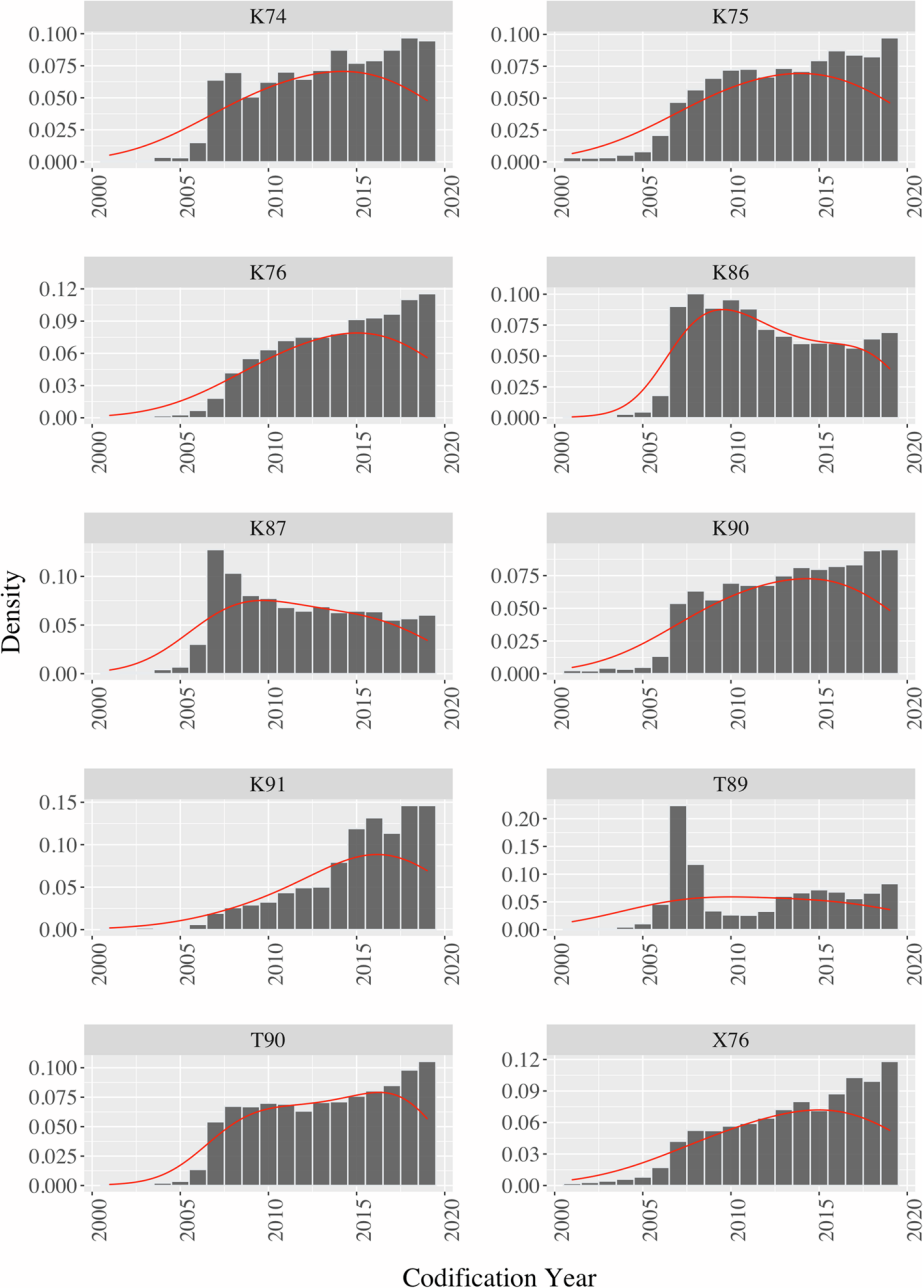
Distribution of other ICPC-2 diagnoses in patients with obesity. K86: Uncomplicated hypertension; T90: Non-insulin-dependent diabetes; K87: Complicated hypertension; K90: Stroke/cerebrovascular accident; X76: Malignant neoplasm breast female; T89: Insulin-dependent diabetes; K75: Acute myocardial infarction; K74: Ischemic heart disease with angina; K76: Ischemic heart disease without angina; K91: Cerebrovascular disease

This study found that the most prevalent morbidities in patients with obesity as coded through ICPC-2 were K86 (uncomplicated hypertension), T90 (non-insulin-dependent diabetes), and K87 (complicated hypertension). These morbidities were still more prevalent when considering the coding before and after obesity coding (supplemental material 2). The distribution of ICPC-2 codifications in patients with obesity is displayed in Fig. 3. Unlike K86 and K87, T90 increased over time.

Uncomplicated hypertension, non-insulin-dependent diabetes, and complicated hypertension were the most prevalent regardless of the obesity class (supplemental material 2).

The most prevalent morbidities in patients with obesity (as per the Microsoft power BI® report data) similarly included uncomplicated hypertension and non-insulin-dependent diabetes, but not the other conditions. In non-T82 individuals, also through the data from the Microsoft power BI® reports, T83 (overweight) was the most common. The other top 10 morbidities included uncomplicated hypertension but not non-insulin-dependent diabetes (T90) (supplemental material 1).

### Use of health resources

Data from the Microsoft power BI® reports also allowed the characterization of the obese population including a comparison with a non-obese population.

**Fig. 4:**
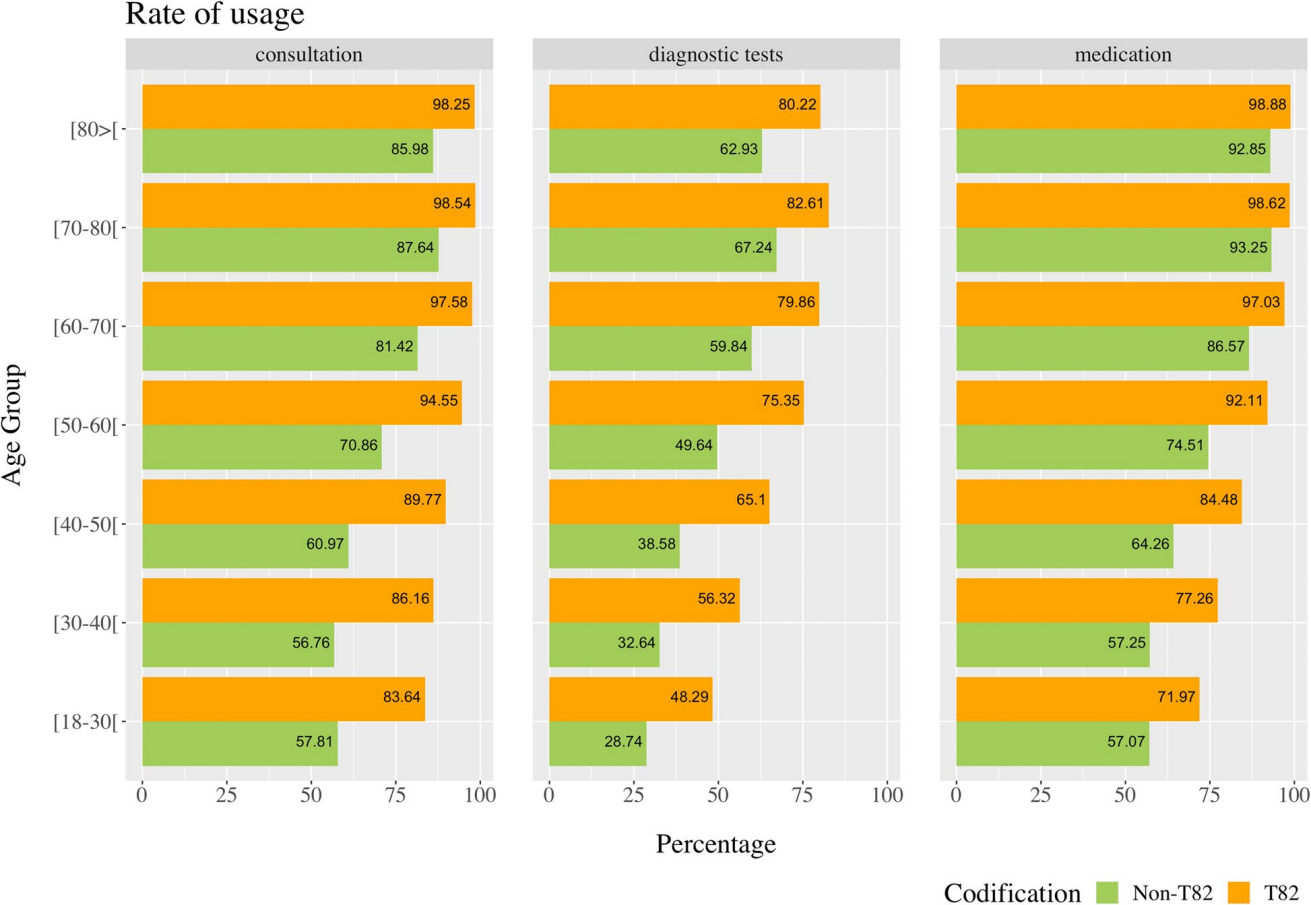
Comparison of usage rates of medical consultations, medications, and diagnostic tests between individuals with and without T82-obesity coding. Non-T82: Without obesity. T82: Obesity

We next compared descriptive information from patients with and without obesity in terms of the use of consultations, medications, and diagnostic tests. T82 subjects always had a higher percentage (Fig. 4). Between 83% (age group of [18–30[ years old) to 99% (age group of [70–80[ years old) of the individuals with obesity have at least one medical consultation per year versus only 57% (age group of [18–30[ years old) to 88% (age group of [70–80[ years old) of the individuals without obesity. This difference is higher, around 29%, in the 30–40-year-old age group. Moreover, the percentage of individuals with obesity that take at least one medication varies between 72% (age group of [18–30[ years old) and 99% (age group of [80 > [ years old) versus the group without obesity which ranges between 57% (age group of [18–30[ years old) and 93% (age group of [70–80[ years old). Once again, a higher difference of 20% was found in the group aged 30–40 and 40–50 years. The subjects without obesity had diagnostic tests use rates of 29% (age group of [18–30[ years old) and 67% (age group of [70–80[ years old), and the group with obesity had values between 48% (age group of [18–30[ years old) and 83% (age group of [70–80[ years old). In this case, the higher difference between the two groups (26%) was found in the group aged between 40–50 and 50–60 years.

**Fig. 5:**
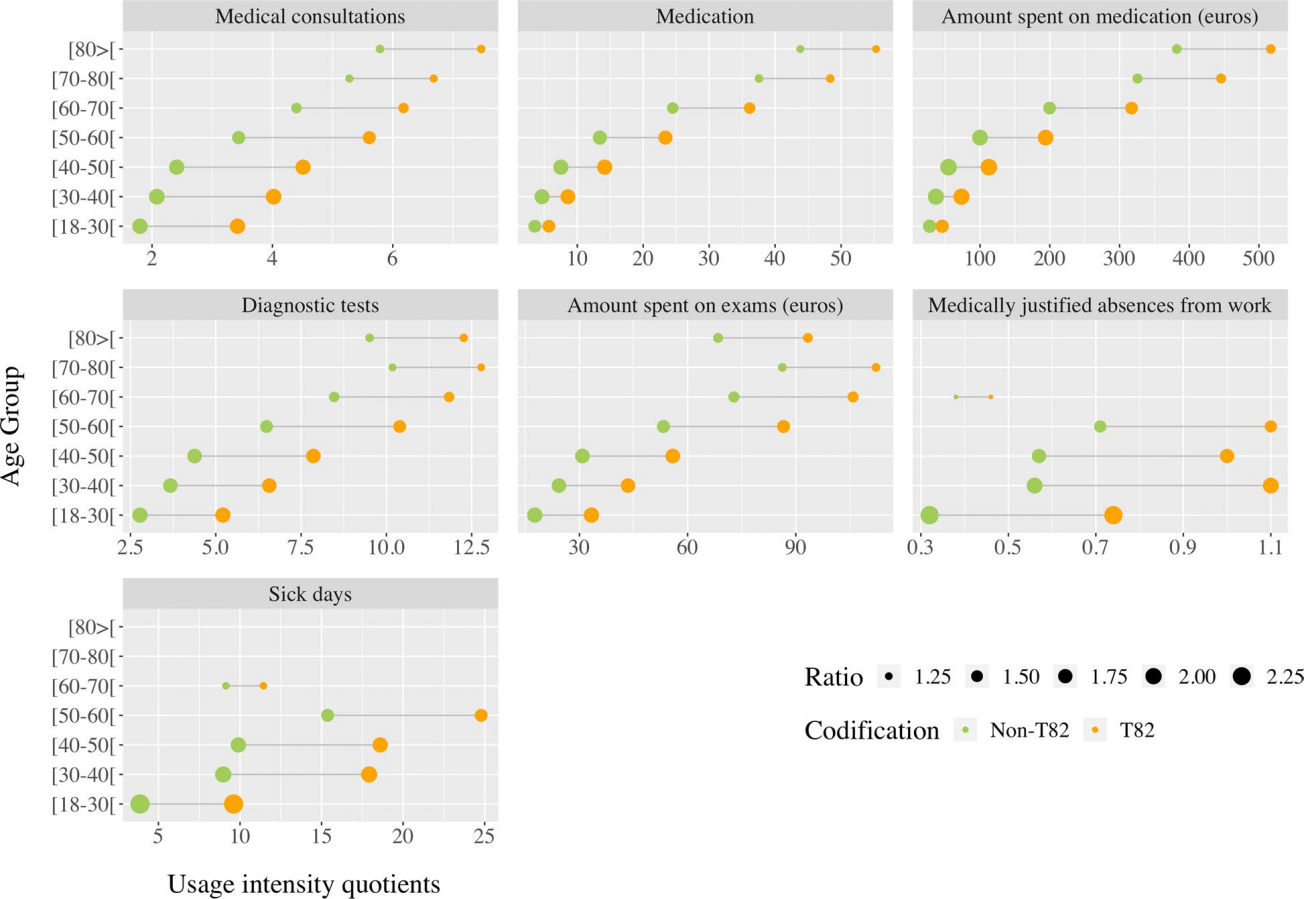
Comparison between T82 and non-T82 including intensity quotients of medical consultations, medications, the amount spent on medication, diagnostic tests, the amount spent on diagnostic tests, medically justified absences from work, and sick days. Non-T82: Without obesity. T82: Obesity

Still considering the comparison between individuals with and without T82 coding, an individual with T82 in the 18–30, 30–40, and 40–50 age groups used almost twice as many consultations as individuals in the same age group and without T82 (83.64, 86.16 and 89.77% versus 57.81, 56,76 and 60.97%, respectively). An individual with T82 in the 30–40 and 40–50 age groups was prescribed more than 1.80 times more medication. The National Health Service spent on that medication, in euros, more than twice of what was spent on those without T82 for the same age groups. Regarding diagnostic tests, this trend toward more diagnostic tests with higher expenses in the T82 group continues. The difference is more expressive in the 18–30, 30–40, and 40–50 age groups with around 1.8-fold more costs per individual. Finally, medically justified absences from work more than doubled in individuals with T82 in the 18–30 age group. An individual with T82 aged 18–30 and 30–40 years at least doubled the number of days of illness versus someone in the same age group without T82. Figure 5 presents a more complete view of this information.

## Discussion

### Summary

The prevalence of obesity was 10.2% in the adult (25–74 years) population of the regional health administration of northern Portugal, in 2019. From a bio-demographic point of view, patients with obesity were predominantly middle-aged women, and obesity class I was seen in 70.0% of the obese population.

Regarding the comorbidity profiles, the most prevalent comorbidities in patients with obesity were uncomplicated hypertension (K86), non-insulin-dependent diabetes (T90), and complicated hypertension (K87). In addition to cardiovascular diseases and diabetes, malignant neoplasm of the female breast (X76) was also identified in the top 10 obesity comorbidities.

Depending on the age group, regarding descriptive information, each individual with T82 used almost twice as many medical consultations and was prescribed 1.80-fold (more than double the expenses for medications). They had about 1.80-fold more expenses with diagnostic tests, needed more than twice as many absences from work for medical reasons, and had at least twice as many sick days.

### Comparison with existing literature

The prevalence of obesity found in the present study (10.2%) was lower than that documented in previous Portuguese studies. The INSEF presented an obesity prevalence of 28.2% (95%CI: 26.4–30.5) for northern Portugal [5]. The National Food, Nutrition, and Physical Activity Survey (IAN-AF) showed an obesity prevalence of 21.6% (95%CI: 19.5–23.8) [18]. Both studies used representative samples of the Portuguese general population but different definitions of the adult population considering individuals aged 25–74 years [5] or 18–64 years [18]. The lower prevalence versus INSEF [5] and IAN-AF [18] is probably due to the real-world data from the EHRs. There might be more patients with obesity in northern Portugal, but they were not included in this study if they are not coded with T82. Supporting this view is the fact that the T82's coding density is significantly higher after 2018 when automatic coding was implemented (Fig. 2).

Proper data recording is another key variable, especially weight, and height. Of the 348 536 individuals, only 279 438 remained after excluding those with values outside the minimum and the maximum of weight and height. Thus, 19.8% of 348 536 individuals were excluded due to inadequate values. This situation raises the suspicion of significant difficulties in the complete assessment of the Portuguese population's BMI through real-world data from EHRs. This emphasizes yet another concern: the difficulty of recognizing obesity as a disease [19] and underdiagnosis that delays the beginning of intervention (lifestyle modifications relating to nutrition and physical activity), which in turn increases the risk of developing morbidities.[3, 20].

The patients with obesity were predominantly women, which is consistent with the INSEF [5] (64.8% vs 58.2%) and the IAN-AF [18] (64.8% vs 57.5%). From a bio-demographic point of view, patients with obesity were predominantly middle-aged women, and obesity class I was found in 70.0% of the obese population. Regarding the distribution of T82 individuals among age groups, the subjects aged 55–64 years had the highest value: 27.2%.

There are no other similar studies for comparison in terms of comorbidities and the use of health resources among Portuguese populations with T82 diagnoses to the best of our knowledge. However, several non-Portuguese studies are consistent with the fact of the most prevalent morbidities associated with obesity are cardiovascular diseases, diabetes, and some cancers like breast cancer [3, 21–23].

Of all morbidities (except T89 (insulin-dependent diabetes)), the coding density of uncomplicated hypertension (K86) and complicated hypertension (K87) appeared to reduce over time, whilst coding of other conditions tended to increase.

An interpretation for these data could be that the higher initial coding density of K86 and K87 is due to the presence of more individuals on these dates already diagnosed (but not with codification) with hypertension than with other morbidities. Current coding density values for hypertension may thus be more real and congruent with the true incidence of hypertension. The earlier awareness of the diagnosis of hypertension—given the impact of cerebrovascular events on Portuguese mortality—may be another reason for this aspect.

In 2015, 3 327 children and young people up to the age of 19 had type 1 diabetes (T89), which corresponded to 0.16% of the Portuguese population [24]. These data suggest that the T89 density graph does not show values ​​in accordance with reality. The values probably reflect a practical coding problem due to the confusion between the concept of "insulin-dependent diabetes" (T89 or type 1 diabetes) and "insulin-treated diabetes" (type 2 diabetes, correctly coded as T90).

A very interesting study on the burden of obesity-related diseases in the WHO European Region (Portugal not included) stated that a 1% reduction in BMI would result in a 4.7% reduction in the prevalence of type 2 diabetes. Even better, a 5% reduction in population BMI would reduce the prevalence of type 2 diabetes by 16.7% across Europe [6] Substantial reductions in all-cause healthcare costs were observed as early as 1 year after sustained weight loss in adults with obesity [25] In a multicenter randomized trial with a 24-month follow-up in primary care, patients with a motivational lifestyle change intervention achieved a 5% or greater reduction from baseline weight, which is a common criterion for clinically significant weight loss [26].

It is not surprising that obesity is related to the greater use of health services and resources. Consistent with previous research, this study demonstrated that T82-coded individuals were prescribed over 1.80-fold more medications with more than double the expense to the National Health Service on medication. A German population-based health survey also stated that patients with obesity are more than twice as likely to have medication prescriptions compared to their healthy-weight counterparts and that the annual costs of individuals with obesity could exceed the costs of individuals with a normal weight by two-fold [27] A large, population-based study in Israel used comprehensive EHR data to show that healthcare costs increased by more than double among individuals with obesity [28].

A recently released study in the Centre for the Study of Evidence-Based Medicine (CEMBE) of the Faculty of Medicine of the University of Lisbon and by the consultant Evigrade-IQVIA showed that treating obesity-related diseases (diabetes, stroke, ischemic heart disease, and chronic kidney disease) costs 88 times more than the cost of treating obesity ‘per se*’*. In 2018, there were 46 269 deaths from obesity-related diseases, which represents 43% of the total deaths that occurred in mainland Portugal that year [29].

The costs of obesity to society are also higher due to absences from work due to illness and employment difficulties [30]. Here, T82-coded individuals had more than twice as many absences from work for medical reasons and had at least twice as many sick days.

Depending on the age group, descriptive information showed that each T82-coded individual used almost twice as many medical consultations and had about 1.8-fold more expenses with diagnostic tests. The greater use of services such as the number of consultations was previously associated with an increased BMI. However, a greater number of consultations did not seem to positively impact the patients' BMI [20]. This situation is probably related to the fact that these consultations are only (or predominantly) aimed at addressing comorbidities arising from obesity and not obesity per se. Comprehensive weight management solutions that integrate lifestyle interventions can be valuable to patients, employers, and payers [25].

The substantial and multi-domain costs associated with obesity emphasize the need for investment to address this important public health problem. Here, there is even a question of proper allocation of resources, which are always scarce and being channeled to deal with the consequences of preventable disease.

### Strengths and limitations

This is the first Portuguese study to use the content of real-world EHRs to study patients with obesity in northern Portugal, representing a pragmatic strategy to understand the impact of obesity. After applying the selection criteria, this real-world database produced a dataset that the authors consider to be of good quality, which appears to be a challenge in real-world studies. By studying this population and having found differences between those with the ICPC-2 T82 code and those without the ICPC-2 T82 code, an important factual value can be recognized about the impact of obesity on the population and health indicators.

The main limitation of this retrospective study is the fact that there may be more patients with obesity in northern Portugal who were not coded as such in primary health care and were not included in this study. Prior to version 2.7, of the electronic information system (March 2019), manual obesity coding was required by the physician. In particular, the lack of valid values ​​for weight and/or height on real-world EHRs implied the direct elimination of an important proportion of individuals. Another limitation of this work is the fact that not all of the subjects with obesity are NHS users. Thus, more accurately, the prevalence estimate presented here will correspond more to the adult (25–74 years) prevalence of obesity among NHS users, in the northern region of Portugal, in 2019. On the other hand, there may be individuals without T82 coding who, despite being so coded, may not have obesity. In summary, the risk of misclassification bias must be admitted.

Eventually, individuals who have more comorbidities (and therefore probably older) may be more likely to have more up-to-date data, as they will have more consultations and, therefore, more opportunities to update their clinical records.

Furthermore, the fact that we did not know exactly whether obesity is coded or not when the patient meets diagnostic criteria greatly limits the temporal analysis of comorbidities in relation to the diagnosis of diabetes.

### Implications for practice and research

This study provides extremely relevant conclusions to Portugal and has real-world data on obesity not previously studied including prevalence, characterization, most prevalent comorbidities, and use of resources and health services. The real-world data comparisons between T82 and non-T82 individuals showed the impact of obesity.

EHRs have evolved making it necessary to understand the quality of documented data to continuously optimize its content and the strategies that depart from it. Thus, routine recording of weight and height deserves special attention to allow obesity (and overweight) recognition at an early stage and move on to the appropriate intervention for this assessment and management. Future work is necessary to automate the codification of obesity for subjects under 18 years of age (a situation that does not yet happen in Portugal). This will be critical to raise awareness of the problem at an earlier stage while anticipating intervention and amplifying their impact in the prevention of problems associated with obesity.

Practical strategies need to be implemented such as creating a health program consultation for obesity for truly targeted approaches to obesity. For this to work, it is necessary to incorporate automated prompts/programmes into EHRs, reimburse medications, train professionals, and provide resources for the support and proper practice of lifestyle medicine.

## Supplementary Information

**Additional file 1. Table S1.** Distribution of the T82 and Non-T82 individuals for age group, through the data from Microsoft power BI® reports. **Table S2.** Distribution of the TOP 10 ICPC-2 codifications in individuals with and without T82 ICPC-2 codification, through the data from Microsoft power BI® reports.

**Additional file 2. Table S3.** Distribution of the TOP 10 ICPC-2 codifications, before and after T82 ICPC-2 codification. *N* = 266 872. **Table S4.** Distribution of the TOP 10 ICPC-2 codifications, before and after T82 ICPC-2 codification, in individuals with obesity class I. *N* = 186 742. **Table S5.** Distribution of the TOP 10 ICPC-2 codifications, before and after T82 ICPC-2 codification, in individuals with obesity class II. *N* = 59 510. **Table S6.** Distribution of the TOP 10 ICPC-2 codifications, before and after T82 ICPC-2 codification, in individuals with obesity class III. *N* = 20 620.

## Data Availability

Data are available upon reasonable request to the corresponding author (Rosália Páscoa). The data processing was carried out through the Department of Studies and Planning of the Regional Health Administration of northern Portugal (Ministry of Health, Portugal). The data were extracted from the server through an anonymized data processing and editing platform and delivered securely (and following legal regulations and due approval) to the principal investigator. The data are property of the Regional Health Administration of northern Portugal (Ministry of Health, Portugal). Data analysis was authorized for the purposes of the protocol only.

## References

[1] World Health Organization (2021). Obesity and overweight: fact sheet.

[2] Guh DP, Zhang W, Bansback N, Amarsi Z, Birmingham CL, Anis AH (2009). The incidence of co-morbidities related to obesity and overweight: A systematic review and meta-analysis. BMC Public Health.

[3] Pantalone KM, Hobbs TM, Chagin KM, Kong SX, Wells BJ, Kattan MW (2017). Prevalence and recognition of obesity and its associated comorbidities: cross-sectional analysis of electronic health record data from a large US integrated health system. BMJ Open.

[4] Weir CB, Jan A (2021). BMI Classification Percentile and Cut Off Points. StatPearls.

[5] Gaio V, Antunes L, Namorado S, Barreto M, Gil A, Kyslaya I (2018). Prevalence of overweight and obesity in Portugal: Results from the First Portuguese Health Examination Survey (INSEF 2015). Obes Res Clin Pract.

[6] Webber L, Divajeva D, Marsh T, McPherson K, Brown M, Galea G (2014). The future burden of obesity-related diseases in the 53 WHO European-Region countries and the impact of effective interventions: a modelling study. BMJ Open.

[7] Bordowitz R, Morland K, Reich D (2007). The use of an electronic medical record to improve documentation and treatment of obesity. Fam Med.

[8] Roth C, Foraker RE, Payne PRO, Embi PJ (2014). Community-level determinants of obesity: harnessing the power of electronic health records for retrospective data analysis. BMC Med Inform Decis Mak.

[9] Baer HJ, Cho I, Walmer RA, Bain PA, Bates DW (2013). Using Electronic Health Records to Address Overweight and Obesity. Am J Prev Med.

[10] Banerjee ES, Gambler A, Fogleman C (2013). Adding obesity to the problem list increases the rate of providers addressing obesity. Fam Med.

[11] Ministério da Saúde. Missão para os Cuidados de Saúde Primários. Linhas de Acção Prioritária para o Desenvolvimento dos Cuidados de Saúde Primários. 2006. Available from: https://www.sns.gov.pt/wp-content/uploads/2016/02/Linhas-de-Acao-Prioritaria-para-o-Desenvolvimento-dos-CSP.pdf

[12] Rocha PD, Sá AB (2011). Reforma da Saúde Familiar em Portugal: avaliação da implantação. Ciência & Saúde Coletiva..

[13] World Organization of National Colleges, Academies, and Academic Associations of General Practitioners/Family Physicians, editor. ICPC-2-R: international classification of primary care. Rev. 2nd ed. Oxford ; New York: Oxford University Press; 2005. 193 p. (Oxford medical publications).

[14] Comissão de Classificações da Organização Mundial de Ordens, Nacionais, Academias e Associações Académicas de Clínicos Gerais/Médicos, de Família (WONCA). Classificação Internacional de Cuidados de Saúde Primários Segunda Edição. 2011. Available from: http://www2.acss.min-saude.pt/Portals/0/apmcg_ICPC%20v%201.7.pdf

[15] World Organization of Family Doctors WONCA (2004). An Introduction to the International Classification of Primary Care Version 2.

[16] Bilhete de Identidade dos Indicadores dos Cuidados de Saúde Primários. Available from: https://bicsp.min-saude.pt/pt/Paginas/default.aspx

[17] Nunes B, Barreto M, Gil AP, Kislaya I, Namorado S, Antunes L (2019). The first Portuguese National Health Examination Survey (2015): design, planning and implementation. J Public Health.

[18] Oliveira A, Araújo J, Severo M, Correia D, Ramos E, by the IAN-AF Consortium (2018). Prevalence of general and abdominal obesity in Portugal: comprehensive results from the National Food, nutrition and physical activity survey 2015–2016. BMC Public Health..

[19] Kyle TK, Dhurandhar EJ, Allison DB (2016). Regarding Obesity as a Disease. Endocrinol Metab Clin North Am.

[20] Mattar A, Carlston D, Sariol G, Yu T, Almustafa A, Melton G (2017). The prevalence of obesity documentation in Primary Care Electronic Medical Records: Are we acknowledging the problem?. Appl Clin Inform.

[21] Finer N (2015). Medical consequences of obesity. Medicine.

[22] Wiebe N, Stenvinkel P, Tonelli M (2019). Associations of chronic inflammation, insulin resistance, and severe obesity with mortality, myocardial infarction, cancer, and chronic pulmonary disease. JAMA Netw Open.

[23] Jehan S, Zizi F, Pandi-Perumal SR, McFarlane SI, Jean-Louis G, Myers AK (2020). Energy imbalance: obesity, associated comorbidities, prevention, management and public health implications. Adv Obes Weight Manag Control.

[24] Sociedade Portuguesa de Diabetologia (2019). Diabetes: Factos e Números – O Ano de 2015 − Relatório Anual do Observatório Nacional da Diabetes – Parte I.

[25] Ding Y, Fan X, Blanchette CM, Smolarz BG, Weng W, Ramasamy A (2021). Economic value of nonsurgical weight loss in adults with obesity. JMCP.

[26] Rodriguez-Cristobal JJ, Alonso-Villaverde C, Panisello JM, Travé-Mercade P, Rodriguez-Cortés F, Marsal JR (2017). Effectiveness of a motivational intervention on overweight/obese patients in the primary healthcare: a cluster randomized trial. BMC Fam Pract.

[27] Teuner CM, Menn P, Heier M, Holle R, John J, Wolfenstetter SB (2013). Impact of BMI and BMI change on future drug expenditures in adults: results from the MONICA/KORA cohort study. BMC Health Serv Res.

[28] Reges O, Leibowitz M, Hirsch A, Dicker D, Finer N, Haase CL (2020). A comprehensive descriptive assessment of obesity related chronic morbidity and estimated annual cost burden from a population-based electronic health record database. Isr J Health Policy Res.

[29] Centre for the Study of Evidence-Based Medicine (CEMBE) of the Faculty of Medicine of the University of Lisbon and by the consultant Evigrade-IQVIA. The Cost and Burden of Overweight and Obesity in Portugal. Available from: https://www.expatica.com/pt/news/obesity-costs-portugal-1-2-billion-euros-a-year-103471/

[30] McPherson K (2008). Does preventing obesity lead to reduced health-care costs?. PLoS Med.

