# Supplementary material for: Characterization of an obese population: a retrospective longitudinal study from real-world data in northern Portugal
Source: BMC Prim Care. 2023 Apr 15;24:99. doi: 10.1186/s12875-023-02023-7 (PMC10105387; doi:10.1186/s12875-023-02023-7)
Supplement: Supplementary file 1 — Additional file 1. Table S1. Distribution of the T82 and Non-T82 individuals for age group, through the data from Microsoft power BI® reports. Table S2. Distribution of the TOP 10 ICPC-2 codifications in individuals with and without T82 ICPC-2 codification, through the data from Microsoft power BI® reports. [file 12875_2023_2023_MOESM1_ESM.pdf]

Supplemental material 1.

Characterization of the obese population **through the data from the Microsoft power BI® reports**.

Table S1. Distribution of the T82 and Non-T82 individuals for age group, through the data from Microsoft power BI® reports.

**Age group**	T82<br>N = 421 149			**Age group**	Non-T82<br>N = 2 883 026	
	n	%			n	%
[18-30[	28 337	5.4		[18-30[	542 985	18.8
[30-40[	39 814	7.9		[30-40[	474 925	16.5
[40-50[	72 207	11.8		[40-50[	540 513	18.8
[50-60[	92 161	15.7		[50-60[	490 126	17.0
[60-70[	92 788	18.9		[60-70[	386 920	13.4
[70-80[	64 635	18.9		[70-80[	265 386	9.2
[80>[	31 207	13.4		[80>[	182 171	6.3

Table S2. Distribution of the TOP 10 ICPC-2 codifications in individuals with and without T82 ICPC-2 codification, through the data from Microsoft power BI® reports.

**ICPCs**	T82<br>N = 421 149			**ICPCs**	Non-T82<br>N = 2 883 026	
	n	%			n	%
T93	204 507	48.6		T83	824 340	28.6
K86	186 033	44.2		T93	738 924	25.6
T90	82 216	19.5		K86	522 679	18.1
L86	81 293	19.3		P17	481 218	16.7
P76	77 223	18.3		A98	453 308	15.7
L90	69 233	16.4		W11	382 786	13.3
K95	54 290	12.9		P76	334 366	11.6
P17	51 960	12.3		L86	311 688	10.8
W11	50 120	11.9		R74	252 331	8.7
P06	48 984	11.6		P74	237 696	8.2

ICPC: International Classification of Primary Care. **T93**: Lipid disorder; **K86**: Hypertension uncomplicated; **T90**: Diabetes non-insulin-dependent; **L86**: Back syndrome with radiating pain; **P76**: Depressive disorder; **L90**: Osteoarthrosis of knee; **K95**: Varicose veins of leg; **P17**: Tobacco abuse; **W11**: Contraception oral; **P06**: Sleep disturbance; **T83**: Overweight; **A98**: Health maint/ preventive medicine; **R74**: Upper respiratory infection acute; **P74**: Anxiety disorder/anxiety state.
