# Supplementary material for: Characterization of an obese population: a retrospective longitudinal study from real-world data in northern Portugal
Source: BMC Prim Care. 2023 Apr 15;24:99. doi: 10.1186/s12875-023-02023-7 (PMC10105387; doi:10.1186/s12875-023-02023-7)
Supplement: Supplementary file 2 — Additional file 2. Table S3. Distribution of the TOP 10 ICPC-2 codifications, before and after T82 ICPC-2 codification. N = 266 872. Table S4. Distribution of the TOP 10 ICPC-2 codifications, before and after T82 ICPC-2 codification, in individuals with obesity class I. N = 186 742. Table S5. Distribution of the TOP 10 ICPC-2 codifications, before and after T82 ICPC-2 codification, in individuals with obesity class II. N = 59 510. Table S6. Distribution of the TOP 10 ICPC-2 codifications, before and after T82 ICPC-2 codification, in individuals with obesity class III. N = 20 620. [file 12875_2023_2023_MOESM2_ESM.pdf]

Supplemental material 2.

Table S3. Distribution of the TOP 10 ICPC-2 codifications, before and after T82 ICPC-2 codification. N = 266 872.

**ICPCs**	Before T82 N = 176009			**ICPCs**	After T82 N = 60209	
	n	%			n	%
K86	103657	38.84		K86	24586	9.21
T90	37663	14.11		T90	19527	7.32
K87	13098	4.91		K87	4608	1.73
K90	3503	1.31		K90	1409	0.53
X76	2666	1.00		X76	1333	0.50
T89	2501	0.94		K76	1224	0.46
K75	2232	0.84		K91	1112	0.42
K74	2014	0.75		T89	1076	0.40
K76	1955	0.73		K74	1038	0.39
K91	1332	0.50		K75	794	0.30

ICPC: International Classification of Primary Care. **K86**: Hypertension uncomplicated; **T90**: Diabetes non-insulin-dependent; **K87**: Hypertension complicated; **K90**: Stroke/ cerebrovascular accident; **X76**: Malignant neoplasm breast female; **T89**: Diabetes insulin-dependent; **K75**: Acute myocardial infarction; **K74**: Ischaemic heart dis with angina; **K76**: Ischaemic heart disease without angina; **K91**: Cerebrovascular disease.

Regarding the three categories of obesity (obesity class I (BMI 30 to 34.9 kg/m^2), obesity class II (BMI 35 to 39.9 kg/m^2) and, obesity class III (BMI greater than or equal to 40 kg/m^2), the distribution of the TOP 10 codifications of other health problems, before and after the obesity codification date, was as referred in Tables S4-S5. The codifications K86: Hypertension uncomplicated, T90: Diabetes non-insulin-dependent, and K87: Hypertension complicated were also the most prevalent, despite the obesity class.

Table S4. Distribution of the TOP 10 ICPC-2 codifications, before and after T82 ICPC-2 codification, in individuals with obesity class I. N = 186 742.

**ICPCs**	Before T82 N = 126526			**ICPCs**	After T82 N = 35866	
	n	%			n	%
K86	73830	39.54		K86	14510	7.77
T90	27054	14.49		T90	11297	6.05
K87	9155	4.90		K87	2633	1.41
K90	2618	1.40		K90	860	0.46
X76	1980	1.06		K76	834	0.45
T89	1682	0.90		X76	777	0.42
K75	1788	0.96		K91	734	0.39
K74	1583	0.85		K74	724	0.39
K76	1543	0.83		T89	609	0.33
K91	1019	0.55		K75	531	0.28

ICPC: International Classification of Primary Care. **K86**: Hypertension uncomplicated; **T90**: Diabetes non-insulin-dependent; **K87**: Hypertension complicated; **K90**: Stroke/ cerebrovascular accident; **X76**: Malignant neoplasm breast female; **T89**: Diabetes insulin-dependent; **K75**: Acute myocardial infarction; **K74**: Ischaemic heart dis with angina; **K76**: Ischaemic heart disease without angina; **K91**: Cerebrovascular disease.

Table S5. Distribution of the TOP 10 ICPC-2 codifications, before and after T82 ICPC-2 codification, in individuals with obesity class II. N = 59 510.

**ICPCs**	Before T82 N = 37772			**ICPCs**	After T82 N = 35866	
	n	%			n	%
K86	22695	38.14		K86	6957	11.69
T90	8146	13.69		T90	5602	9.41
K87	2923	4.91		K87	1342	2.26
K90	693	1.16		K90	400	0.67
T89	585	0.98		X76	380	0.64
X76	549	0.92		K76	312	0.52
K75	363	0.61		T89	301	0.51
K74	348	0.58		K91	283	0.48
K76	333	0.56		K74	237	0.40
K91	253	0.43		K75	196	0.33

ICPC: International Classification of Primary Care. **K86**: Hypertension uncomplicated; **T90**: Diabetes non-insulin-dependent; **K87**: Hypertension complicated; **K90**: Stroke/ cerebrovascular accident; **X76**: Malignant neoplasm breast female; **T89**: Diabetes insulin-dependent; **K75**: Acute myocardial infarction; **K74**: Ischaemic heart dis with angina; **K76**: Ischaemic heart disease without angina; **K91**: Cerebrovascular disease.

Table S6. Distribution of the TOP 10 ICPC-2 codifications, before and after T82 ICPC-2 codification, in individuals with obesity class III. N = 20 620.

**ICPCs**	Before T82 N = 11711			**ICPCs**	After T82 N = 7479	
	n	%			n	%
K86	7132	34.59		K86	3119	15.13
T90	2463	11.94		T90	2628	12.74
K87	1020	4.95		K87	633	3.07
K90	192	0.93		X76	176	0.85
X76	137	0.66		T89	166	0.81
T89	234	1.13		K90	149	0.72
K75	81	0.39		K91	95	0.46
K74	83	0.40		K76	78	0.38
K76	79	0.38		K74	77	0.37
K91	60	0.29		K75	67	0.32

ICPC: International Classification of Primary Care. **K86**: Hypertension uncomplicated; **T90**: Diabetes non-insulin-dependent; **K87**: Hypertension complicated; **K90**: Stroke/ cerebrovascular accident; **X76**: Malignant neoplasm breast female; **T89**: Diabetes insulin-dependent; **K75**: Acute myocardial infarction; **K74**: Ischaemic heart dis with angina; **K76**: Ischaemic heart disease without angina; **K91**: Cerebrovascular disease.

Note: The analysis of these aggregated data refers to the records as of 12.31.2019 and, therefore, after the implementation of the T82 automated coding.
